# Altered anxiety-related and abnormal social behaviors in rats exposed to early life seizures

**DOI:** 10.3389/fnbeh.2013.00036

**Published:** 2013-05-09

**Authors:** Adelisandra Silva Santos Castelhano, Gustavo dos Santos Teada Cassane, Fulvio Alexandre Scorza, Roberta Monterazzo Cysneiros

**Affiliations:** ^1^Laboratory of Neurobiology, Developmental Disabilities Graduate Program, Presbyterian Mackenzie UniversitySão Paulo, Brazil; ^2^Department of Neurology and Neurosurgery, Experimental Neurology, Escola Paulista de Medicina, Federal University of São PauloSão Paulo, Brazil

**Keywords:** neonatal *status epilepticus*, pilocarpine, social anxiety, general anxiety, social behavior

## Abstract

Neonatal seizures are the most common manifestation of *neurological dysfunction in the neonate*. The prognosis of neonatal seizures is highly variable, and the controversy remains whether the severity, duration, or frequency of seizures may contribute to brain damage independently of its etiology. Animal data indicates that seizures during development are associated with a high probability of long-term adverse effects such as learning and memory impairment, behavioral changes and even epilepsy, which is strongly age dependent, as well as the severity, duration, and frequency of seizures. In preliminary studies, we demonstrated that adolescent male rats exposed to one-*single* neonatal *status epilepticus* (SE) episode showed social behavior impairment, and we proposed the model as relevant for studies of developmental disorders. Based on these facts, the goal of this study was to verify the existence of a persistent deficit and if the anxiety-related behavior could be associated with that impairment. To do so, male Wistar rats at 9 days postnatal were submitted to a single episode of SE by pilocarpine injection (380 mg/kg, i.p.) and control animals received saline (0.9%, 0.1 mL/10 g). It was possible to demonstrate that in adulthood, animals exposed to neonatal SE displayed low preference for social novelty, anxiety-related behavior, and increased stereotyped behavior in anxiogenic environment with no locomotor activity changes. On the balance, these data suggests that neonatal SE in rodents leads to altered anxiety-related and abnormal social behaviors.

## Introduction

*Status epilepticus* (SE), an acute condition characterized by repetitive or prolonged seizures, affects between 120,000 to 200,000 people per year in the United States (Neill et al., 2005; Lowenstein, 2006). It occurs more often in children than in adults, and 40–50% of children under the age of 2 years (Shinnar et al., 1997). The incidence of neonatal seizures varies according to the age, severity of the etiologic factor, and of the population studied. The clear incidence has not yet been established, although it has been estimate that varies between 1.1–8.6/1000 live births (Saliba et al., 1996, 1999; Sheth et al., 1999; Mosley, 2010). Furthermore, the prognosis of neonatal seizures is highly variable (Costa et al., 2001), and persists the controversy whether the severity, duration or frequency of seizures may contribute to brain damage independently of its etiology (Udani, 2008). In general terms, half of the cases exhibit minimum sequels and the other half evolves into death or serious squeals (Costa et al., 2001). In addition, newborns with transient focal ischemia, metabolic abnormalities without a clear etiology seem to evolve in a more satisfactory manner, unlike those with hypoxic-ischemic encephalopathy, infection of the nervous system and prenatal brain dysgenesis that generally have a worse prognosis (Udani, 2008). From a clinical standpoint, most studies fail to show that seizures *per se* regardless the severity of the etiologic factor is able to disrupt brain development. In this context, animal models allow the observation and analysis of variables under controlled conditions and have been used to explore these issues. Thus, the animal models with neonatal injury that results in permanent impairment, especially of social interaction are considered useful for the study of neurodevelopmental disorders, such as schizophrenia and autism (Schneider and Koch, 2005; Tordjman et al., 2007).

Following these lines of reasoning, animal data indicates that seizures during development are associated with a high probability of long-term adverse effects such as, learning and memory impairment, behavioral changes and epilepsy that are strongly age dependent, as well as the severity, duration, and frequency of seizures (Holmes, 2004, 2009). The mechanisms that underlie these changes are not yet completely understood, but neonatal seizures do not lead to cell loss, rather than considerable synaptic reorganization. Studies focused primarily on behavioral changes, rather than epileptogenic activity, are less frequent. Two studies have shown that neonatal seizures produce anxiety-related behavior (Sayin et al., 2004; Shi et al., 2007), and another study demonstrated deficit of cognitive flexibility (Kleen et al., 2011). Recently, we demonstrated that the neonatal SE (Castelhano et al., 2010) leads to impairment of social interaction in adolescent male rats. Our study brings an important contribution to demonstrate that sequels of neonatal seizures extending beyond the learning and memory deficits, increased anxiety and loss in cognitive flexibility. Animals studies reveal that early life seizures can disrupt some brain structure such as the hippocampus (Holmes et al., 1998; Lynch et al., 2000; Santos et al., 2000; Villeneuve et al., 2000; Sogawa et al., 2001; Cornejo et al., 2007; Nishimura et al., 2011) neocortex (da Silva et al., 2005; Isaeva et al., 2010), prefrontal cortex (PFC) (Kleen et al., 2011) and thalamus (Santos et al., 2000; Kubová et al., 2001) allowing the argument that the behavioral implications are broad, impacting the cognitive, and adaptive performance. In human condition, different neonatal conditions are likely to cause neurological damage e alter brain development. Atladóttir et al. (2012) using cohort study design investigated neonatal conditions and the risk for Autism Spectrum Disorders (ASDs). They found an increased risk of ASD after exposure to perinatal hypoxia, neonatal seizures, intracranial hemorrhage, neonatal hypoglycemia, neonatal septicemia, and meningitis.

Based on accumulated evidence, animal models submitted to neonatal seizures may be relevant for studies of neurodevelopmental disorders. In this context, it is necessary a more comprehensive study on the behavioral repertoire, particularly in the area of sociability, repetitive behaviors, and anxiety. On the balance, the aim of the present study was to investigate the existence of a persistent deficit and if the anxiety-related behavior could be associated with that impairment.

## Materials and methods

### Animals

All procedures were approved by Universidade Presbiteriana Mackenzie Ethical Committee (CEUA, 076/02/2011). Newly born Wistar rats were maintained under controlled conditions (07:00–19:00 h, light/dark cycle; 22–24°C) with their mother. Pups' ages were determined from the day of birth (P0). Colonies were randomly assigned into different groups. All procedures were carried out in male rats.

### SE induction

SE is usually defined as continuous seizure activity lasting for 30 min or longer or intermittent seizures lasting 30 min or more from which the patient does not regain consciousness (Commission on Classification and Terminology of the International League Against Epilepsy—ILAE, 1989). However, it has been suggested that seizure activity lasting for 20 min could also be qualified as SE (Lowenstein et al., 1999). Experimental group (*n* = 40) received pilocarpine 3.8% in saline (380 mg/kg, i.p), on P9 which corresponds to a full-term neonate (Holopainen, 2008), and control group (*n* = 30) received saline solution instead pilocarpine (0.1 mL/10 g). SE started within 3–4 min following pilocarpine injection being characterized by continuous intense body tremor, scratching, clonic movements of forelimbs and head bobbing. Following cessation of SE (ca 4 h) animals returned to their mothers. The rate of mortality in experimental group after SE induction was about 37%, yielding 25 animals. At 21 days postnatal, 2–3 male animals of each litter were randomly chosen, housed together (4–5 animals per cage) and distributed in the following groups:

- Experimental group (EXP), 16 male rats submitted to neonatal SE.
- Control group (CTR), 14 male rats that received saline injection.

The reminiscent animals, being 9 for EXP and 16 for CTR were perfused and/or decapitated to further studies.

### Testing procedure

The behavioral tests started from 60 days postnatal and were videotaped. Only male offspring were investigated. The home cage containing the animals was transferred to the testing room 60 min before each day session. All apparatus were cleaned with a 5% alcohol solution after each behavioral procedure. All behavioral tests were carried out in the same room with a controlled intensity of light (9 l×). At the end of behavioral tests, for both groups, half of the animals were perfused and the others had their brains removed and frozen for further studies. This procedure was adopted to avoid replicate experiments, reducing the use of animals and finally to allow the correlation between data.

#### Sociability

The social behavior apparatus was adapted by Novaes et al. (2012) from Crawley (2007). The apparatus was an acrylic rectangular box divided into three compartments of equal size (39 cm height × 26 cm width × 41 cm deep) by retractable doors. The sociability test, preceded by a habituation period in the apparatus, was divided in three sequential phases of the 10 min each. During the habituation period, the test rat was placed in the middle chamber for 10 min with the retractable doors closed. Each of the two sides contained an identical empty wire cage. After the habituation period, the retractable doors were opened allowing the test rat to explore the entire apparatus. In this phase, the number of entries and time spent in each compartment with objects were measured. In the social approach phase, an unfamiliar rat was enclosed in one of the wire cages and a human observer scored the number of snout-snout contacts between the test rat and the unfamiliar rat. In the social novelty phase, a new unfamiliar rat was enclosed into the wire cage in the opposite compartment and all parameters were again investigated. Of important, before the introduction of a social stimulus, the test rest was trapped in the central chamber.

#### Elevated plus-maze

The elevated plus-maze is based on a natural tendency of the animals to explore a new environment vs. the aversive properties of an elevated open runway. The apparatus had two closed arms with walls 45 cm in height and two open arms 50 cm long (Insight Ltda, Brazil). The maze was elevated 50 cm from the floor. The animals were placed in the center of the maze with their nose pointing toward an open arm and allowed to freely explore the maze for 10 min. The sections were videotaped, and the number of entries and the time spent in both arms were recorded. Both parameters were expressed as percent of entries or time in open arms [(Open arm/Open arm + Closed arm) × 100].

#### Open field

The apparatus consisted of a circular arena (100 cm diameter) enclosed by plain white walls and a floor divided into 12 zones, being 8 peripheral and 4 central (Insight Ltda, Brazil). Each animal was placed into the central area and observed for 10 min. During this time, the locomotor activity was expressed as the number of peripheral, central, or total lines crossed. Central locomotion was also expressed as ratio of central to total locomotion. In addition, the time of immobility and number of grooming episodes were measured. The test was repeated 7 days later, in order to investigate habituation after repeated exposure to the same stimulus. Repeated exposure to the open field apparatus results in time dependent changes in behavior.

### Statistical analyses

The data were expressed as mean ± standard error. The sociability test was analyzed by Mixed ANOVA using condition (object 1 × object 2, object × unfamiliar rat or familiar rat vs. social novelty) as within-subjects factor and groups (EXP vs. CTR) as between-subjects factor (Kohl et al., 2013). Significant effects were probed with *post-hoc* testing (Bonferroni). The Open Field's parameters were analyzed by Mixed ANOVA, using Bonferroni for *post-hoc* testing. The elevated plus maze's parameters were analyzed by Student's *T*-test for independent samples. *p* values of 0.05 or less were considered significant. The analyses were effectuated using commercial program (Prism 5.03 for windows).

## Results

### Sociability

**Figure 1:**
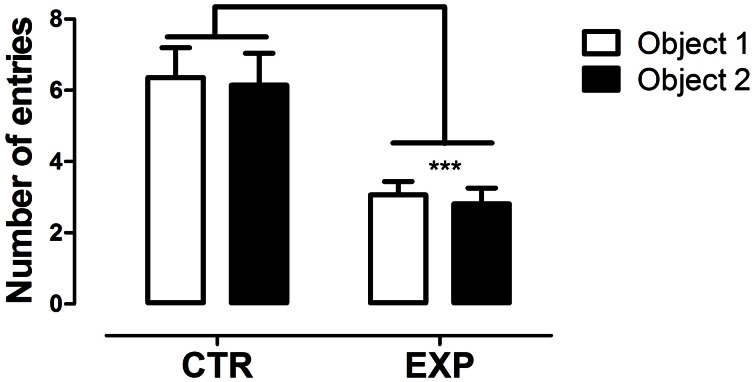
**Number of entries into compartments with objects is shown as mean ± standard error.** Control group (CTR) *n* = 14 and Experimental group (EXP), *n* = 16. The number of entries between compartments did not differ for both groups, but EXP exhibited less number of entries into both compartments. ^***^p < 0.0001.

In the first test phase, the number of entries between the compartments with objects did not differ for both groups [EXP and CTR, *F*_(1.28)_ = 0.81; NS], but EXP exhibited less number of entries as compared to CTR for both compartments [*F*_(1.28)_ = 13.96, *p* < 0.008], Figure 1. In addition, no effect of interaction was noted [*F*_(1.28)_ = 4.00; NS] for time spent in the compartments. In this phase, the groups did not reveal preference for any compartments with objects, although the EXP has showed less motor activity.

**Figure 2:**
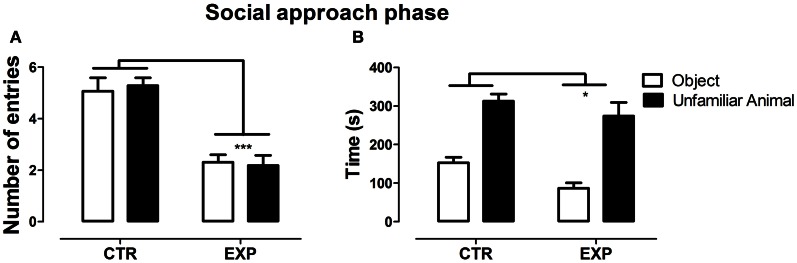
**Number of entries (A) and time spent (B) into social (rat) and non-social (object) compartments is shown as mean ± standard error.** Control group (CTR) *n* = 14 and Experimental group (EXP), *n* = 16. EXP showed less locomotor activity compared to CTR (^***^p < 0.0001), but both groups spent more time on the side with unfamiliar rat.

In the social approach phase, with the introduction of an unfamiliar rat into one of the compartments, significant difference was observed between groups for number of entries into the compartments [*F*_(1.28)_ = 33.96, *p* < 0.0001], but no effect was observed neither for compartments [*F*_(1.28)_ = 0.051; NS], nor for interaction [*F*_(1.28)_ = 0.74; NS]. EXP exhibited less number of entries as compared to CTR for both compartments, Figure 2A. In parallel, both groups spent more time in the compartment with unfamiliar rat as compared with object [*F*_(1.28)_ = 62.97, *p* < 0.0001], but EXP spent less time than CTR in both compartments [*F*_(1.28)_ = 4.69, ^*^p < 0.038], Figure 2B. In this phase, experimental group demonstrated being as interested as the control for social encounter.

**Figure 3:**
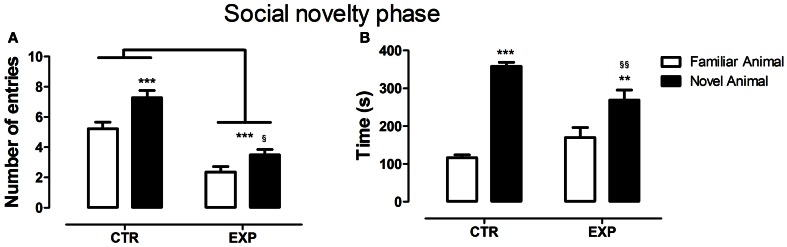
**Number of entries (A) and time spent (B) into compartments with familiar and novel rat is shown as mean ± standard error.** Control group (CTR) *n* = 14 and Experimental group (EXP), *n* = 16. In **(A)**, less number of entries was noted to the EXP (^***^p < 0.0001), but both groups exhibited higher number of entries into compartments with unfamiliar rat (EXP, *t* = 2.65, ^§^*p* < 0.05; CTR, *t* = 4.46, ^***^p < 0.001). In **(B)**, the CTR showed a clear preference for the social novelty spending more time with an unfamiliar rat than did the EXP (^§§^*p* < 0.01, ^**^p < 0.01).

**Figure 4:**
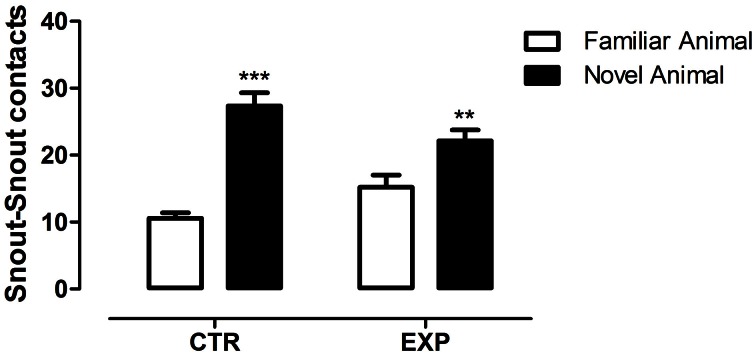
**Number of snout-snout contacts is shown as mean ± standard error.** Control group (CTR), *n* = 14 and Experimental group (EXP), *n* = 16. EXP exhibited less preference for the social novelty than did CTR. ^**^p < 0.01, ^***^p < 0.0001.

In the social novelty phase, with an introduction of a novel rat into another wire cage, the differences between groups became more evident. Under this condition, a significant difference was observed between groups for number of entries into compartments [*F*_(1.28)_ = 46.52, *p* < 0.0001] and between compartments [*F*_(1.28)_ = 26.48, *p* < 0.0001] with no effect of interaction [*F*_(1.28)_ = 2.32, NS]. Both groups exhibited higher number of entries into compartments with unfamiliar rat (EXP, *t* = 2.65, ^§^*p* < 0.05; CTR, *t* = 4.46, ^***^p < 0.001), Figure 3A. As the locomotion was highly different between groups, number of entries was reanalyzed as ratio of novel to familiar. The ratio was significantly less in EXP as compared to CTR (1.25 ± 0.25 vs. 1.54 ± 0.4, respectively, *t* = 2.37, *p* = 0.025). For time spent with familiar and novel rat, a significant effect of interaction was noted [*F*_(1.28)_ = 10.95; *p* < 0.0026], indicating that EXP exhibited a decreased preference for social novelty, Figure 3B. Finally, for snout-snout contacts a significant effect of interaction was noted for the factors compartments vs. group, [*F*_(1.28)_ = 10.08, *p* = 0.0036], Figure 4. CTR exhibited more preference for the social novelty than did EXP.

In summary, statistical analysis revealed that animals exposed to neonatal SE showed less preference for social novelty. Additionally, the animals also exhibited reduced locomotor activity which could be less motivation or an anxiogenic-like effect of exposure to social stress. In next step, we evaluated the locomotor activity and anxiety-like behavior.

### Elevated plus-maze (EPM)

**Figure 5:**
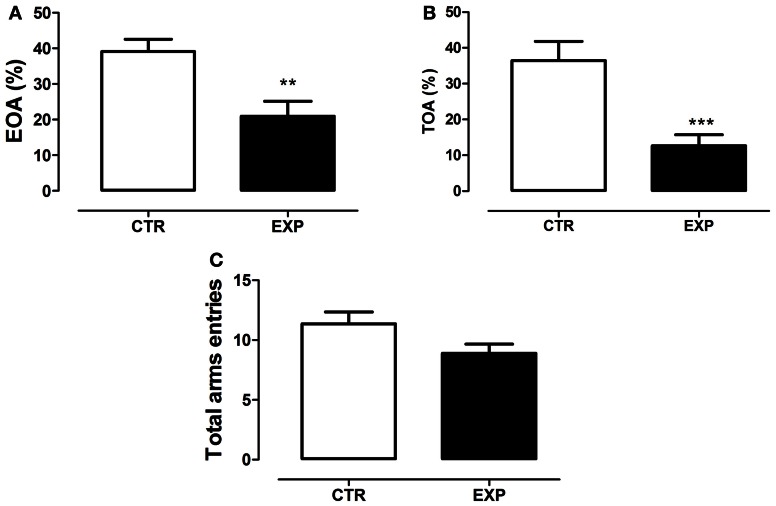
**Percentage of entries onto the open arms (A), percentage of time spent on the open arms (B) and total entries in both arms (C) are shown as mean ± standard error.** Control group (CTR) *n* = 14 and Experimental group (EXP), *n* = 16. EXP exhibited less entries **(A)** and time **(B)** on the open arms as compared to CTR group (^***^p = 0.0008 and ^**^p = 0.001). In **(C)**, the locomotor activity was only marginally different between groups (*p* = 0.055).

The statistical analysis revealed a significant difference between groups on anxiety-like behavior. EXP exhibited less entries (*t* = 3.29, *df* = 28, *p* = 0.0027, Figure 5A) and time on the open arms (*t* = 3.95, *df* = 28, *p* = 0.0005, Figure 5B). The locomotor activity was only marginally different between groups (*t* = 2.003, *df* = 28, *p* = 0.055, Figure 5C). In summary, the statistical analysis revealed that the neonatal SE induced anxiety-like behavior.

### Open field

**Figure 6:**
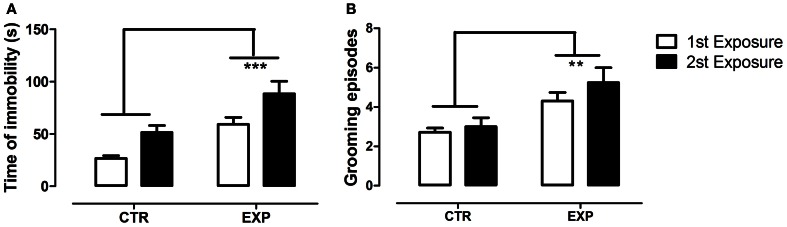
**Time of immobility (A) and number of grooming episodes (B) are shown as mean ± standard error.** Control group (CTR) *n* = 14 and Experimental group (EXP), *n* = 16. The EXP exhibited higher time of immobility **(A)** and grooming episodes **(B)** as compared to CTR, ^**^p = 0.0037, ^***^p = 0.0005, respectively.

**Figure 7:**
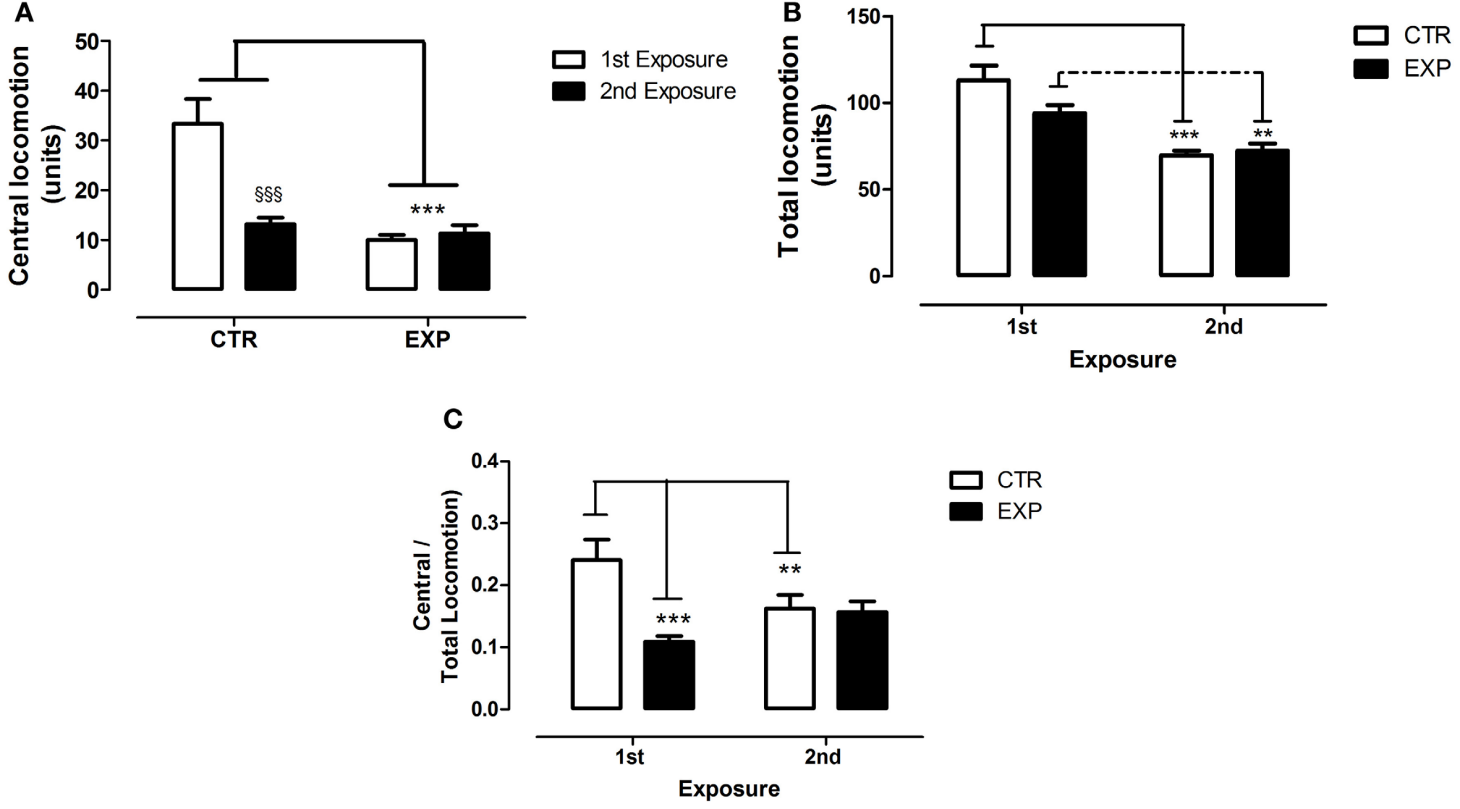
**Central (A), total locomotion (B) and (C) ratio of central to total locomotion are shown as mean ± standard error in both exposures.** Control group (CTR) *n* = 14 and Experimental group (EXP), *n* = 16. Reduced central locomotion was noted for EXP in both exposures. In CTR, the locomotor activity in central squares decreased significantly during second exposure **(A)**. The total locomotion did not differ between groups, but decreased significantly during 2nd exposure **(B)**. In **(C)**, the ratio of central to total locomotion was significantly less in EXP as compared to CTR, decreased on the 2nd exposure for CTR and was kept unchanged for EXP. ^**^p < 0.001, ^***^p < 0.0001 and ^§§§^*p* < 0.0001.

To the time of immobility, Two-Way ANOVA revealed a significant effect of group [EXP vs. CTR, *F*_(1.28)_ = 15.421, *df* = 28; *p* = 0.0005] and of the exposure [1st × 2nd, *F*_(1.28)_ = 13.71; *df* = 28; *p* = 0.0009], and no effect of interaction [*F*_(1.28)_ = 0.103, *df* = 28; *p* = 0.749]. Thus, EXP exhibited higher time of immobility as compared to CTR in both exposures, Figure 6A. To grooming episodes, EXP exhibited more number of events [*F*_(1.28)_ = 10.026; *df* = 28; *p* = 0.0037] as compared to CTR, Figure 6B, and no effects of exposure [*F*_(1.28)_ = 2.14, *df* = 28; *p* = 0.154] nor of interaction were noted [*F*_(1.28)_ = 0.609, *df* = 28; *p* = 0.441]. These effects were accompanied by reduction of central locomotor activity as compared to CTR [*F*_(1.28)_ = 27.72; *df* = 28; *p* < 0.0001]. In addition, it was noted effect of exposure [*F*_(1.28)_ = 14.02; *df* = 28; *p* < 0.0001] and interaction between factors [*F*_(1.28)_ = 18.23; *df* = 28; *p* < 0.0002]. In the first exposure, central locomotion was significantly less in EXP comparatively to CTR (*t* = 6.26, ^§§§^*p* < 0.001), and kept unchanged in the second exposure, Figure 7A. However, the total locomotion did not differ between groups [*F*_(1.28)_ = 1.8921; *df* = 28; *p* = 0.1799], Figure 7B, but decreased on 0second exposure for both [*F*_(1.28)_ = 48.05; *df* = 28; *p* < 0.0001], suggesting long term habituation to a novel environment. Taking in account that EXP exhibited a general tendency for decreased locomotion, and in order to magnify the interpretation of results, it was analyzed the ratio of central locomotion to total locomotion. The ratio was significantly lower in EXP as compared to CTR [*F*_(1.28)_ = 6.89; *df* = 28; *p* = 0.013] and effect of interaction was also observed [*F*_(1.28)_ = 12.78; *df* = 28; *p* = 0.012]. The ratio significantly decreased on the first exposure in the EXP as compared to CTR (*t* = 6.26, ^***^p < 0.001), and in the CTR on second exposure (*t* = 3.32. ^**^p < 0.01), but was kept unchanged for experimental group. EXP, which exhibited high level of anxiety at first exposure to central zone, kept the same behavior at second exposure, suggesting no habituation to anxiogenic environment, Figure 7C.

The behavioral data of the open field revealed that neonatal SE increased emotionality of rats when exposed to a new environment, especially when it offers greater risk of attack or exposition, as the central area of the open field. It was also observed increased of grooming episodes and the time of immobility with no alterations in general locomotor activity.

## Discussion

In the present study we used a three—chambers choice test to assess social behavior, elevated plus maze and open field to evaluated general anxiety. Adult animals exposed to neonatal SE showed less preference for social novelty, increased emotionality in an unfamiliar and threatening environment, increased grooming episodes and immobility, reduced locomotor activity in situations of higher risk. Taken as a whole, the data suggest that early life seizures in rats leads to increased emotionality and abnormal social behavior.

The social interaction test developed by File and Hyde (1978) was the first to measure experimental anxiety based on ethologic concept. Posteriorly, Haller and Bakos (2002) proposed a novel animal model of anxiety in a social context (Social Approach-Avoidance test). In this paradigm, prestressed Wistar rats spent significantly less time as compared to control in a social compartment containing a unfamiliar rat confined behind a transparent wall, demonstrating the anxiogenic-like effect of the stress on approach-avoidance behavior (Nicolas and Prinssen, 2006). In the sociability paradigm, the animals exposed to neonatal SE displayed less preference for social novelty than controls. We previously demonstrated that pilocarpine-induced neonatal SE impaired social play behavior (Castelhano et al., 2010). It is well-established that adolescence is an important period for the development of adult social competence and that an inadequate social development, in addition to seizures itself or maladaptive response following seizures, may contribute to social anxiety in adulthood. In rodents, higher levels of social anxiety can disrupt the preference between two stimuli in a preference test (Mines et al., 2010). This effect could be related to decreased motivation, increased emotionality or impaired social memory. Lin et al. (2009) reported in lithium-pilocarpine-induced neonatal seizures reduced levels of dopamine in the PFC. It is well-established that the dopamine found in the PFC originates from the ventral tegmental area, a structure involved in neural network of social behaviors (Goodson and Kabelik, 2009) is also involved in and emotional responses (Baskerville and Douglas, 2010). In this sense, dopamine reduction in PFC could lead to less engagement in social interactions. However, taking in account that in social approach phase experimental group demonstrated being as interested as the control for a social encounter, the probable mechanisms to explain reduced preference for social novelty are the increased state of anxiety associate with the deficit in social discrimination. There is emerging in the literature a discussion about the bi-directional relationship and possibly cyclic, between social interaction impairment and the degree of anxiety or emotionality. Anxiety intensifies social impairment, and poor social function contributes to anxiety (Nicolas and Prinssen, 2006; White et al., 2010). In this sense, animals were evaluated in the open field and the elevated plus-maze. Experimental animals avoided the central area of the open field in both exposures, suggesting no habituation to anxiogenic environment, but they performed similarly to control in total locomotion suggesting no locomotor activity changes. In addition, they displayed higher immobility and stereotyped behavior. In EPM, they spent less time and reduced number of entries on the open arms, suggesting the presence of general anxiety. Reduced locomotor activity on sociability test, even after the habituation period and before social presence, suggest that the new environment increased the state of anxiety and that no habituation to anxiogenic space was reached, as observed in the central zone of the open field. Rodents also have emotional behaviors similar to humans when exposed to specific experimental situations (Ramos and Mormède, 1998). In this regard, Nicolas and Prinssen (2006) evaluated the social approach-avoidance behavior in a high-anxiety strain, F-344 rats. The animals exhibited spontaneous avoidance behavior that was sensitive to benzodiazepine agonist and inverse agonist in a bidirectional manner. To the best of our knowledge, we are the first group to demonstrate abnormal social behavior following early life seizures. However, general anxiety was previously reported. Corroborating with our results, Sayin et al. (2004) demonstrated anxiety-related behavior without locomotor activity changes in adult animals submitted to SE by kainic acid injection during early stage of development. Oliveira et al. (2007) reported anxiety-like behavior in adult rats submitted to lithium-pilocarpine model at 15 days postnatal. The enhanced emotionality could be associated to changes in hippocampus function. Lugo et al. (2012) and Benedikt et al. (2012) demonstrated that hippocampal damage leads to anxiety-like behavior. Beyond of the hippocampus, others brain regions exhibit maladaptive response following neonatal seizures. For example, Kleen et al. (2011) observed that adults male Wistar rats aged between 1 and 10 days postnatal that undergo a series to of neonatal seizures by fluorothyl administration exhibited structural changes of the PFC that were positively correlated with the impairment of cognitive flexibility and insistence to maintain a routine previously learned. Individuals with autism often have repetitive behaviors and strong resistance to the change of routine. Failure to change routine in rodents may be analogous to the cognitive inflexibility. In stressful situations is common the emergence or intensification of stereotypes. In our study, we observed increase in self-grooming when animals were exposed to an anxiogenic environment. The association of grooming behavior with anxiety or stress is well-established in animal models. Stressful events or environments (Langen et al., 2011) as well as appetitive situations can cause an animal to develop abnormal repetitive behavior (Spruijt et al., 1992; Van Erp et al., 1994). Additionally, the enhanced stereotyped behavior could be also due of imbalance both within and between the motor, cognitive and limbic corticostriatal circuits. The corticostriatal circuits include striatum, globus pallidus, substancia nigra, thalamus, and cortex. It is well-established that corticostriatal circuits are functionally divided into three circuits related to the predominant cerebral cortical input to striatum: sensoriomotor circuit functionally related to movements (comprising the motor and oculomotor loops), associative circuit functionally related to cognitive functions (dorsolateral prefrontal loop) and the limbic circuit functionally related emotional-motivational behavior (lateral orbitofrontal and anterior cingulate loops) (for an extensive review see; Langen et al., 2011). Abnormal repetition of behavior can result from damage to any of the corticostriatal circuits. Experimental data demonstrated that SE can damage brain regions involved with corticostriatal circuit. Fernandes et al. (1999) observed hypermetabolism in cortical and forebrain regions plus the substantia nigra following lithium–*pilocarpine*-induced SE in immature rats. Kubová et al. (2001) demonstrated the presence of silver-positive cells predominantly in the mediodorsal, but also too in the ventrolateral and ventromedial thalamic nucleus in rats that experienced pilocarpine-induced SE at the age of 12 days postnatal. Interestingly, the mediodorsal and ventrolateral nucleus of thalamus are part of limbic and sensoriomotor circuit, respectively. Taking in account that stereotypies can function as coping mechanism to reduce animal's arousal level when it is exposed to stressful events or environments (Langen et al., 2011), it is licit to suppose that animals with damaged thalamus nuclei could be more prone to experience stereotypies under these situations.

As previously mentioned, the decreased preference for social novelty could be also associated to inability of animals exposed to neonatal SE to distinguish a familiar from an unfamiliar conspecifics. In support to this hypothesis, Sankar et al. (1998) reported in rats submitted to pilocarpine-induced seizures during second week of life, damage in the amygdala and hippocampus, brain regions implicated in social recognition memory an emotionality (Ferguson et al., 2002; Yang et al., 2013). Thus, the abnormal social behavior noted in animals exposed to early life seizures could be related to high state of anxiety and impaired social memory.

Taken as a whole, adult animals exposed to neonatal SE displayed less preference for social novelty than control, anxiety-related behavior, increased stereotyped behavior in anxiogenic environment without locomotor activity changes. The data suggest that neonatal SE in rodents leads to altered anxiety-related and abnormal social behaviors.

### Conflict of interest statement

The authors declare that the research was conducted in the absence of any commercial or financial relationships that could be construed as a potential conflict of interest.

## References

[B1] Atladóttir H. O. Schendel D. E. Lauritsen M. B. Henriksen T. B. Parner E. T. (2012). Patterns of contact with hospital for children with an autism spectrum disorder: a Danish register-based study. J. Autism Dev. Disord. 42, 1717–1728 10.1007/s10803-011-1416-5 22160299

[B2] Baskerville T. A. Douglas A. J. (2010). Dopamine and oxytocin interactions underlying behaviors: potential contributions to behavioral disorders. CNS Neurosci. Ther. 16, 92–23 10.1111/j.1755-5949.2010.00154.x 20557568 PMC6493805

[B3] Benedikt J. Inyushin M. Kucheryavykh Y. V. Rivera Y. Kucheryavykh L. Y. Nichols C. G. (2012). Intracellular polyamines enhance astrocytic coupling. Neuroreport 5, 23, 1021–1025 10.1097/WNR.0b013e32835aa04b 23076119 PMC3658138

[B4] Castelhano A. S. Scorza F. A. Teixeira M. C. Arida R. M. Cavalheiro E. A. Cysneiros R. M. (2010). Social play impairment following status epilepticus during early development. J. Neural Transmiss. 117, 1155–1160 10.1007/s00702-010-0460-1 20711791

[B5] Cornejo B. J. Mesches M. H. Coultrap S. Browning M. D. Benke T. A. (2007). A single episode of neonatal seizures permanently alters glutamatergic synapses. Ann. Neurol. 61, 411–426 10.1002/ana.21071 17323345

[B6] Costa J. C. Nunes M. L. Fiori R. M. (2001). Convulsões no período neonatal. J. Pediatr. 77, S1–S115 10.2223/jped.225 14676899

[B7] Crawley J. N. (2007). Mouse behavioral assays relevant to the symptoms of autism. Brain Pathol. 17, 448–459 10.1111/j.1750-3639.2007.00096.x 17919130 PMC8095652

[B8] da Silva V. A. Regondi M. C. Cavalheiro E. A. Spreafico R. (2005). Disruption of cortical development as a consequence of repetitive pilocarpine-induced Status epilepticus in rats. Epilepsia 46, 22–30 10.1111/j.1528-1167.2005.01003.x 15987249

[B9] Ferguson J. N. Young L. J. Insel T. R. (2002). The neuroendocrine basis of social recognition. Front. Neuroendocrinol. 23:229 10.1006/frne.2002.0229 11950245

[B10] Fernandes C. González M. I. Wilson C. A. File S. E. (1999). Factor analysis shows that female rat behaviour is characterized primarily by activity, male rats are driven by sex and anxiety. Pharmacol. Biochem. Behav. 64, 731–738 10.1016/S0091-3057(99)00139-2 10593196

[B11] File S. E. Hyde J. R. (1978). Can social interaction be used to measure anxiety? Br. J. Pharmacol. 62, 19–24 563752 10.1111/j.1476-5381.1978.tb07001.x PMC1667770

[B12] Goodson J. L. Kabelik D. (2009). Dynamic limbic networks and social diversity in vertebrates: from neural context to neuromodulatory patterning. Front. Neuroendocrinol. 30:7 10.1016/j.yfrne.2009.05.007 19520105 PMC2763925

[B13] Haller J. Bakos N. (2002). Stress-induced social avoidance: a new model of stress-induced anxiety? Physiol. Behav. 77, 327–332 10.1016/S0031-9384(02)00860-0 12419409

[B14] Holmes G. L. (2004). Effects of early seizures on later behavior and epileptogenicity. Ment. Retard. Dev. Disabil. Res. Rev. 10, 101–105 10.1002/mrdd.20019 15362164

[B15] Holmes G. L. (2009). The long-term effects of neonatal seizures. Clin. Perinatol. 36, 901–914 10.1016/j.clp.2009.07.012 19944841

[B16] Holmes G. L. Gairsa J. L. Chevassus-Au-Louis N. Ben-Ari Y. (1998). Consequences of neonatal seizures in the rat: morphological and behavioral effects. Ann. Neurol. 44, 845–857 10.1002/ana.410440602 9851428

[B17] Holopainen I. E. (2008). Seizures in the developing brain: cellular and molecular mechanisms of neuronal damage, neurogenesis and cellular reorganization. Neurochem. Int. 52, 935–947 10.1016/j.neuint.2007.10.021 18093696

[B18] Isaeva E. Isaev D. Savrasova A. Khazipov R. Holmes G. L. (2010). Recurrent neonatal seizures result in long-term increases in neuronal network excitability in the rat neocortex. Eur. J. Neurosci. 31, 1446–1455 10.1111/j.1460-9568.2010.07179.x 20384780 PMC3148010

[B19] Kleen J. K. Wu E. X. Holmes G. L. Scott R. C. Lenck-Santini P. P. (2011). Enhanced oscillatory activity in the hippocampal-prefrontal network is related to short-term memory function after early-life seizures. J. Neurosci. 31, 15397–15406 10.1523/JNEUROSCI.2196-11.2011 22031886 PMC3224083

[B20] Kohl C. Riccio O. Grosse J. Zanoletti O. Fournier C. Schmidt M. V. (2013). Hippocampal Neuroligin-2 overexpression leads to reduced aggression and inhibited novelty reactivity in rats. PLoS ONE 8:e56871 10.1371/journal.pone.0056871 23451101 PMC3579928

[B21] Kubová H. Druga R. Lukasiuk K. Suchomelová L. Haugvicová R. Jirmanová I. (2001). Status epilepticus causes necrotic damage in the mediodorsal nucleus of the thalamus in immature rats. J. Neurosci. 21, 3593–3599 11331388 10.1523/JNEUROSCI.21-10-03593.2001 PMC6762492

[B22] Langen M. Kas M. J. Staal W. G. van Engeland H. Durston S. (2011). The neurobiology of repetitive behavior: of mice… Neurosci. Biobehav. Rev. 35, 345–355 10.1016/j.neubiorev.2010.02.004 20156480

[B23] Lin T. C. Huang L. T. Huang Y. N. Chen G. S. Wang J. Y. (2009). Neonatal Status epilepticus alters prefrontal-striatal circuitry and enhances methamphetamine-induced behavioral sensitization in adolescence. Epilepsy Behav. 14, 316–323 10.1016/j.yebeh.2008.12.005 19126440

[B24] Lowenstein D. H. (2006). The management of refractory Status epilepticus: an update. Epilepsia 47, 35–40 10.1111/j.1528-1167.2006.00658.x 17044824

[B25] Lowenstein D. H. Bleck T. Macdonald R. L. (1999). It's time to revise the definition of status epilepticus. Epilepsia 40, 120–122 9924914 10.1111/j.1528-1157.1999.tb02000.x

[B26] Lugo J. N. Brewster A. L. Spencer C. M. Anderson A. E. (2012). Kv4. 2 knockout mice have hippocampal-dependent learning and memory deficits. Learn. Mem. 19, 182–189 10.1101/lm.023614.111 22505720 PMC3348517

[B27] Lynch M. Sayin U. Golarai G. Sutula T. (2000). Long-term consequences of early postnatal seizures on hippocampal learning and plasticity. Eur. J. Neurosci. 12, 2252–2264 10.1046/j.1460-9568.2000.00117.x 10947804

[B28] Mines M. A. Yuskaitis C. J. King M. K. Beurel E. Jope R. S. (2010). GSK3 influences social preference and anxiety-related behaviors during social interaction in a mouse model of fragile X syndrome and autism. PLoS ONE 5:e9706 10.1371/journal.pone.0009706 20300527 PMC2838793

[B29] Mosley M. (2010). Neonatal seizures. Pediatr. Rev. 31, 127–128 10.1542/pir.31-3-127 20194905

[B30] Neill J. C. Liu Z. Mikati M. Holmes G. L. (2005). Pilocarpine seizures cause age-dependent impairment in auditory location discrimination J. Exp. Anal. Behav. 84, 357–350 16596970 10.1901/jeab.2005.84-04 PMC1389772

[B31] Nicolas L. B. Prinssen E. P. (2006). Social approach-avoidance behavior of a high-anxiety strain of rats: effects of benzodiazepine receptor ligands. Psychopharmacology (Berl.) 184, 65–74 10.1007/s00213-005-0233-y 16328377

[B32] Nishimura M. Gu X. Swann J. W. (2011). Seizures in early life suppress hippocampal dendrite growth while impairing spatial learning. Neurobiol. Dis. 44, 205–214 10.1016/j.nbd.2011.07.002 21777677 PMC3167037

[B33] Novaes G. F. Amado D. Scorza F. A. Cysneiros R. M. (2012). Social behavior impairment in offspring exposed to maternal seizures *in utero*. J. Neural Transm. 119, 639–644 10.1007/s00702-011-0751-1 22358065

[B34] Oliveira M. R. Silvestrin R. B. Mello e Souza T. Moreira J. C. (2007). Oxidative stress in the hippocampus, anxiety-like behavior and decreased locomotory and exploratory activity of adult rats: effects of sub acute vitamin A supplementation at therapeutic doses. Neurotoxicology 28, 1191–1199 10.1016/j.neuro.2007.07.008 17727954

[B35] Ramos A. Mormède P. (1998). Stress and emotionality: a multidimensional and genetic approach. Neurosci. Biobehav. Rev. 22, 33–57 10.1016/S0149-7634(97)00001-8 9491939

[B36] Saliba R. M. Annegers J. F. Mizrahi E. M. (1996). Incidence of clinical neonatal seizures. Epilepsia 37, 13

[B37] Saliba R. M. Annegers J. F. Waller D. K. Tyson J. E. Mizrahi E. M. (1999). Incidence of neonatal seizures in Harris County, Texas, 1992–1994. Am. J. Epidemiol. 150, 763–69 10512430 10.1093/oxfordjournals.aje.a010079

[B38] Sankar R. Shin D. H. Liu H. Mazarati A. Pereira de Vasconcelos A. Wasterlain C. G. (1998). Patterns of status epilepticus-induced neuronal injury during development and long-term consequences. J. Neurosci. 18, 8382–8393 9763481 10.1523/JNEUROSCI.18-20-08382.1998 PMC6792849

[B39] Santos N. F. Marques R. H. Correia L. Sinigaglia-Coimbra R. Calderazzo L. Sanabria E. R. (2000). Multiple pilocarpine-induced status epilepticus in developing rats: a long-term behavioral and electrophysiological study. Epilepsia 41, S57–S63 10999521 10.1111/j.1528-1157.2000.tb01558.x

[B40] Sayin U. Sutula T. P. Stafstrom C. E. (2004). Seizures in the developing brain causes adverse long-term effects on spatial learning and anxiety. Epilepsia 45, 1539–1548 10.1111/j.0013-9580.2004.54903.x 15571512

[B41] Schneider M. Koch M. (2005). Deficient social and play behavior in juvenile and adult rats after neonatal cortical lesion: effects of chronic pubertal cannabinoid treatment. Neuropsychopharmacology 30, 944–957 10.1038/sj.npp.1300634 15592349

[B42] Sheth R. D. Hobbs G. R. Mullett M. (1999). Neonatal seizures: incidence, onset, and etiology by gestational age. J. Perinatol. 19, 40–43 10685200 10.1038/sj.jp.7200107

[B43] Shi X. Y. Wang J. W. Lei G. F. Sun R. P. (2007). Long-term effects of recurrent seizures on learning, behavior and anxiety: an experimental study in rats. World J. Pediatr. 3, 61–65

[B44] Shinnar S. Pellock J. M. Moshé S. L. Maytal J. O'Dell C. Driscoll S. M. (1997). In whom does Status epilepticus occur: age-related differences in children. Epilepsia 38, 907–914 9579892 10.1111/j.1528-1157.1997.tb01256.x

[B45] Sogawa Y. Monokoshi M. Silveira D. C. Cha B. H. Cilio M. R. Mccabe B. K. (2001). Timing of cognitive deficits following neonatal seizures: relationship to histological changes in the hippocampus. Brain Res. Dev. Brain Res. 131, 73–83 10.1016/S0165-3806(01)00265-6 11718838

[B46] Spruijt B. M. Van Hooff J. A. Gispen W. H. (1992). Ethology and neurobiology of grooming behavior. Physiol. Rev. 72, 825–852 1320764 10.1152/physrev.1992.72.3.825

[B47] Tordjman S. Drapier D. Bonnot O. Graignic R. Fortes S. Cohen D. (2007). Animal models relevant to schizophrenia and autism: validity and limitations. Behav. Genet. 37, 61–78 10.1007/s10519-006-9120-5 17160702

[B48] Udani V. (2008). Long-term prognosis of neonatal seizures – where are we? Indian Pediatr. 45, 739–741 18820378

[B49] Van Erp A. M. Kruk M. R. Meelis W. Willekens-Bramer D. C. (1994). Effect of environmental stressors on time course, variability and form of self-grooming in the rat: handling, social contact, defeat, novelty, restraint and fur moistening. Behav. Brain Res. 65, 47–55 10.1016/0166-4328(94)90072-8 7880454

[B50] Villeneuve N. Ben-Ari Y. Holmes G. L. Gaiarsa J. L. (2000). Neonatal seizures induced persistent changes in intrinsic properties of CA1 rat hippocampal cells. Ann. Neurol. 47, 729–738 10852538

[B51] White S. W. Albano A. M. Johnson C. R. Kasari C. Ollendick T. Klin A. (2010). Development of a cognitive-behavioral intervention program to treat anxiety and social deficits in teens with high-functioning autism. Clin. Child Fam. Psychol. Rev. 13, 77–90 10.1007/s10567-009-0062-3 20091348 PMC2863047

[B52] Yang L. Zou B. Xiong X. Pascual C. Xie J. Malik A. (2013). Hypocretin/Orexin neurons contribute to hippocampus-dependent social memory and synaptic plasticity in mice. J. Neurosci. 33, 5275–5284 10.1523/JNEUROSCI.3200-12.2013 23516292 PMC3640412

